# Calendar

**DOI:** 10.1155/S146392467900081X

**Published:** 1979


high by 10 cm diameter to be accommodated. The Si (Li) detector assembly is cooled with liquid nitrogen which needs replacing every 12 days. The 4000-channel analyser with dual interlocking bus structure optimises the data collection and processing efficiency. The single keyboard input is provided with a series of dynamic function keys that give step-by-step guidance through the analytical procedures. Results and analytical parameters are presented on a colour video monitor, with a choice of presentation modes.

The modular design of the system enables the PV 9500 to be supplied as a basic qualitative version, or with full quantitative capability.

*N.V. Philip' Gloeilampenfabrieken, Science & Industry, TQ III-2, Eindhoven, The Netherlands.*

### Vacuum emission spectrometer

Philips have introduced a new version of their PV 8350 vacuum emission spectrometer, with a wider range of excitation options that includes inductively coupled plasma (ICP) and glow discharge. ICP permits analysis of any sample that can be presented in aqueous or organic solution, measuring the elements present over wide concentration ranges and down to ultratrace levels. Glow discharge is a more recently developed excitation method that shows great promise for analysis of alloys and study of element concentration gradients as a function of depth within a sample.

The compact new model incorporates stable, fully insulated optics with a simultaneous measurement capacity for up to 40 elements. Operation of the spectrometer is fully automatic, under pre-programmable electronic control. In the basic instrument results are presented as spectral line intensities, recorded on a digital printer. Optional computer packages provide direct read-out of element concentrations and automatic recalibration. This software includes a range of calculation routines and a choice of analytical procedures.

For dedicated process or quality control applications, the spectrometer can be supplied with a single source unit such as the Philips 500 Hz mono-alternace system. Where analytical needs are more varied, the PV 8350 can be equipped with more than one modular source unit, while a specially developed system allows different types of measurement to be interchanged in less than a minute.

*N.V. Philips' Gloeilampenfabrieken, Science & Industry, TQ III-2, Eindhoven, The Netherlands.*

### Calendar

**Editor's Note:**

***Organisers of conferences, seminars etc. should send details for inclusion in this calandar as soon as the relevant information is available and not later than three months before the event.***

### 1979

**Plasma emission spectroscopy**
November 13 – 14, Harrowgate, Yorks
*D.J. Willis, L.R.I.C., Rank Hilger, Westwood Industrial Estate, Ramsgate Rd., Margate, Kent.*

**Computers in the clinical laboratory**
November 19 – 20, Dusseldorf
*Barbara Soltys, Avenue Marnix, l9A, 1050 Brussels, Belgium.*

**Moisture measurement in solids and gases: available techniques and applications**
November 27 – 28, Chislehurst
*Mrs. R.G. Keiller, Sira Institute Ltd., South Hill, Chislehurst, Kent BR7 5EH.*

**Hydrodynamic chromatography**
December 5, London
*J.E.C. Harris, Materials Quality Assurance Directorate, MoD, Puritan, Bridgwater, Somerset.*

**Chemistry in the EEC**
December 11 – 12, London
*Dr. Peter Baker, 6 Poplar Road, Merton Park, London, SW19.*

### 1980

**Developments in atomic plasma spectrochemical analysis**
January 6 – 11, San Juan, Puerto Rico
*ICP Information Newsletter, Chemistry GRC Tower I, University of Massachusetts, Amherst, 01003, USA.*

**Recent advances in organic mass spectrometry in analytical chemistry**
February 6, London
*The Secretary, Analytical Division, Chemical Society, Burlington House, London W1V OBN.*

**Sophistication in instrumentation – has it gone too far?**
February 13, London
*D.J. Willis, L.R.I.C., Rank Hilger, Westwood Industrial Estate, Ramsgate Road, Margate, Kent.*

**Advances in applied high-performance liquid chromatography**
February 20, Edinburgh
*Diana Simpson, Analysis For Industry, Factories 2/3, Bosworth House, High Street, Thorpe-le-Soken, Essex CO16 OEA.*

**The Pittsburgh Conference**
March 10 – 14, Atlantic City NJ
*Dr. R.S. Danchik, 25 Thorncrest Drive, Pittsburgh, PA 15235, USA.*

**International Chromatography Conference**
March 20 – 21, Florida
*Alena Enterprises of Canada, PO Box 1779, Cornwall, Ontario, Canada K6H 5V7.*

**Seventh Annual Report on analytical atomic spectroscopy symposium**
March 27, Sheffield
*D.J. Willis, L.R.I.C., Rank Hilger, Westwood Industrial Estate, Ramsgate Rd, Margate, Kent.*

**Annual Chemistry Congress**
April 9 – 11, Durham
*Dr. J.F. Gibson, The Chemical Society, Burlington House, London W1V OBN.*

**2nd International Congress on Phosphorous Compounds**
April 21 – 25, Boston
*IMPHOS, 8 rue de Pentievre, 75008, Paris, France.*

**Labware 80**
April 22 – 24, London
*Labware Promotions, 28 Worple Road, London SW19 4EE.*

**Analytica 80**
April 29 – May 2, Munich
*ECL (Exhibition Agencies) Ltd., 11 Manchester Square, London W1.*

**Analytical chemistry of pollutants**
May 28 - 30, Dortmund, FRG
*Dr. J. Wendenburg, Gesellschaft Deutscher Chemiker, PO Box 900440, D-6000 Frankfurtam, Main 90, FRG.*

**High speed automatic analysis**
May 30, Glasgow
*D.G. Porter, Laboratory of the Government Chemist, Cornwall House, Stamford Street, London SE1 9NQ.*

**Automation at SAC 80**
July 20 – 26, Lancaster, UK.
*The Secretary, Analytical Division, The Chemical Society, Burlington House, London W1Y OBN.*

**Laboratory 80**
September 9 – 11, London
*Curtis Steadman, 34 – 36 High Street, Saffron Waldon, Essex.*

### 1981

**Euroanalysis IV**
August 23 – 28, Helsinki, Finland,
*Association of Finnish Chemical Societies. Executive Secretary, Pohj, Hesperiankatu 3B10, SF-00260 Helsinki 26, Finland.*

